# Na_3_MgB_37_Si_9_: an icosa­hedral B_12_ cluster framework containing {Si_8_} units

**DOI:** 10.1107/S2056989022000494

**Published:** 2022-01-18

**Authors:** Haruhiko Morito, Takuji Ikeda, Yukari Katsura, Hisanori Yamane

**Affiliations:** a Institute for Materials Research, Tohoku University, 2-1-1 Katahira, Aoba-ku, Sendai 980-8577, Japan; b Research Institute for Chemical Process Technology, National Institute of Advanced Industrial Science and Technology, 4-2-1, Nigatake, Miyagino-ku, Sendai 983-8551, Japan; c National Institute for Materials Science, 1-2-1 Sengen, Tsukuba, Ibaraki, 305-0047, Japan; d Institute of Multidisciplinary Research for Advanced Materials, Tohoku, University, 2-1-1 Katahira, Aoba-ku, Sendai 980-8577, Japan

**Keywords:** crystal structure, boron-rich boride, sodium, silicon, single-crystal, B_2_O_3_ flux, crystal structure, X-ray diffraction

## Abstract

Single crystals of a novel sodium–magnesium boride silicide, Na_3_MgB_37_Si_9_ [*a* = 10.1630 (3) Å, *c* = 16.5742 (6) Å, space group *R*  *m* (No. 166)], were synthesized by heating a mixture of Na, Si and crystalline B with B_2_O_3_ flux in Mg vapor at 1373 K. The Mg atoms in the title compound are located at an inter­stitial site of the Dy_2.1_B_37_Si_9_-type structure with an occupancy of 0.5. The (001) layers of B_12_ icosa­hedra stack along the *c*-axis direction with shifting in the [–*a*/3, *b*/3, *c*/3] direction. A three-dimensional framework structure of the layers is formed *via* B—Si bonds and {Si_8_} units of [Si]_3_—Si—Si—[Si]_3_.

## Abstract 2

Single crystals of a novel boride silicide, Na_3_MgB_37_Si_9_, containing B_12_ icosa­hedra and Si_8_ units were synthesized from Na, B, Si, B_2_O_3_ and magnesium vapor.

## Chemical context

Boron-rich compounds composed of B_12_ icosa­hedral clusters are attracting attention as thermoelectric materials because of their low thermal conductivity resulting from their complicated crystal structures (Cahill *et al.*, 1977 ▸). In our previous study, a novel ternary borosilicide, Na_8_B_74.5_Si_17.5_, was synthesized, and its crystal structure (Morito *et al.* 2010 ▸) and electronic structure measured using soft X-ray spectrometry (Terauchi *et al.* 2018 ▸), have been reported. This compound has a three-dimensional framework structure with layers composed of B_12_ icosa­hedral clusters and Si chains in the channels of the B_12_ clusters. During the investigation of this compound, a new crystalline phase was synthesized in which the stacking sequence of the B_12_ cluster layers differed from that of Na_8_B_74.5_Si_17.5_. The composition analysis revealed that the new phase contained a small amount of Mg derived from an impurity in the starting material of amorphous B powder. Single crystals of this phase were prepared in the present study by heating a starting mixture of Na, crystalline B, a flux of B_2_O_3_ with Mg vapor, and the crystal structure was determined using single-crystal X-ray diffraction.

## Structural commentary

**Figure 1:**
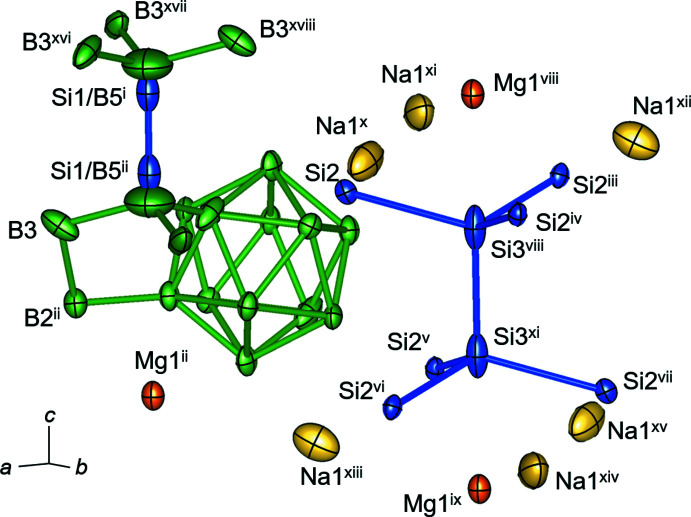
Inter­connection of B_12_ clusters, Si1/B5—Si1/B5 bonds, {Si_8_} units and Na and Mg atoms in Na_3_MgB_37_Si_9_. Displacement ellipsoids are drawn at the 90% probability level. Symmetry codes: (i) *x* + 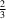 , *y* + 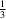 , *z* + 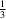 ; (ii) −*x* + 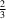 , −*y* + 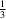 , −*z* + 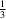 ; (iii) −*x* + *y*, 1 − *x*, *z*; (iv) 1 − *y*, 1 + *x* − *y*, *z*; (v) *y* − 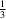 , −*x* + *y* + 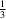 , −*z* + 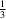 ; (vi) *x* − *y* + 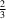 , *x* + 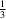 , −*z* + 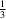 ; (vii) −*x* + 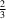 , −*y* − 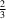 , −*z* + 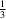 ; (viii) −*x* + 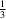 , −*y* + 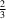 , −*z* + 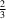 ; (ix) *x* + 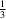 , *y* + 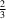 , *z* − 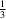 ; (*x*) −*y* + 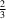 , *x* − *y* + 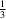 , *z* + 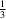 ; (xi) *x* − 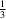 , *y* + 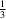 , *z* + 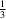 ; (xii) −*x* + *y* + 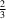 , −*x* + 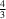 , *z* + 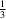 ; (xiii) 1 − *x* + *y*, 1 − *x*, *z*; (xiv) *x*, 1 + *y*, *z*; (xv) −*y*, *x* − *y*, *z*; (xvi) *x* − *y* + 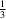 , *x* − 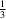 , −*z* + 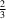 ; (xvii) *y* + 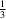 , −*x* + *y* + 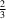 , −*z* + 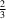 ; (xviii) −*x* + 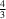 , −*y* + 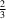 , −*z* + 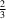 .

The crystal structure of the new phase of composition Na_3_MgB_37_Si_9_ is trigonal (space group *R* 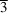 *m*, No. 166), and the hexa­gonal lattice constants are *a* = 10.1630 (3) Å and *c* = 16.5742 (6) Å. The structure is composed of B_12_ icosa­hedral clusters: the B—B distances of the 30 distinct bonds in the cluster are in the range of 1.791 (3)–1.843 (5) Å and the average distance is 1.811 Å (Table 1 ▸). The B_12_ icosa­hedral clusters are connected by a B2—B2 bond [1.761 (5) Å] on the (001) plane and form layers that stack along the *c* axis with a sequence of *ABCABC* by shifts of [–*a*/3, *b*/3, *c*/3] (Figs. 1 ▸ and 2 ▸).

Six B_12_ units in the layers surround {Si_8_} units of composition [Si2]_3_—Si3—Si3—[Si2]_3_. The bond lengths of 2.304 (3) Å for Si3—Si3 and 2.3951 (9) Å for Si2—Si3 are comparable with the bond length in crystalline silicon (2.35 Å). The bond angles of Si2—Si3—Si2 and Si2—Si3—Si3 are 113.86 (3)° and 104.61 (4)°, respectively, which are distorted from the regular tetra­hedral bond angle of 109.47°. The Si2—B1 distance is 2.043 (2) Å, which is close to the Si—B distances (1.973–2.027 Å) found in β-silicon boride, SiB_3_ (Salvador *et al.* 2003 ▸).

The framework structure of B_12_ icosa­hedra and {Si_8_} units of the title compound has also been reported in the structures of Mg_3_B_36_Si_9_C (Ludwig *et al.* 2013 ▸), *RE*_1–*x*_B_12_Si_3.3–*δ*_ (*RE* = Y, Gd–Lu) (0 ≤ *x* ≤ 0.5, *δ* ∼ 0.3) (Zhang *et al.* 2003 ▸) and *RE*_1-*x*_B_36_Si_9_C (*RE* = Y, Gd–Lu) (Ludwig *et al.* 2013 ▸) with the same space group of *R* 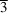 *m*. The {Si_8_} units with Si2—B4 bonds [2.082 (3) Å] and Si1/B5—Si1/B5 pairs that bind to the B atoms at B3 connect the B_12_ layers of Na_3_MgB_37_Si_9_ (Fig. 1 ▸). Because the Si1—Si1 distance of 1.460 (10) Å is short for an Si—Si bond and the B5—B5 distance 2.47 (4) Å is long for a B—B bond, it was concluded that disordered pairs of Si1—B5 and B5—Si1 [B—Si = 1.96 (2) Å] are statistically present with equal occupancies. Similar disordered Si/B—Si/B pairs have been reported in Dy_0.7_B_12.33_Si_3_ (Si/B occupancy 0.5/0.5, Si—B length = 1.838 Å; Zhang *et al.* 2003 ▸). Instead of Si/B—Si/B pairs (Ludwig *et al.* 2013 ▸), Mg_3_B_36_Si_9_C contains Si/C—Si/C pairs (Si/C occupancy 0.507/0.493, Si—C length = 1.881 Å).

The Na1 site in the title compound is located around the {Si_8_} unit between the B_12_ cluster layers. The Na1—Si2 distance is 2.8620 (4) Å and the Na1—B1 and Na1—B2 distances are 2.811 (2) and 2.793 (2) Å, respectively. These distances are almost the same as the Na—Si distance of Na_4_Si_4_ [2.878 (3) Å; Morito *et al.*, 2015 ▸] and Na—B distance of NaB_15_ (2.798 Å; Naslain & Kasper, 1970 ▸). The Mg1 atom is situated above and below the {Si_8_} unit along the *c*-axis direction with an occupancy of 0.5. The Mg1—Si3 and Mg1—B2 distances are 2.403 (4) Å and 2.333 (3) Å, respectively, which are close to the Mg—Si (2.436 Å) and Mg—B distances (2.353 Å) in MgB_12_Si_2_ (Ludwig & Hillebrecht, 2006 ▸). The Na1—Mg1 distance in the title compound is 3.0389 (9) Å, which is close to the Na—Mg distance (3.120 Å) reported in Na_4_Mg_4_Sn_3_ (Yamada *et al.* 2015 ▸). The site corresponding to the location of Mg1 in the title compound does not exist in Mg_3_B_36_Si_9_C (Ludwig *et al.* 2013 ▸), *RE*_1-*x*_B_12_Si_3.3-*δ*_ (*RE* = Y, Gd–Lu) (0 ≤ *x* ≤ 0.5, *δ* ∼ 0.3) (Zhang *et al.* 2003 ▸) and *RE*_1-*x*_B_36_Si_9_C (*RE* = Y, Gd–Lu) (Ludwig *et al.* 2013 ▸).

The number of electrons provided from Na and Mg to the framework of B_37_Si_9_ is five in Na_3_MgB_37_Si_9_. In related compounds, the Mg atom in Mg_3_B_36_Si_9_C and the Dy atom in Dy_0.7_B_12.33_Si_3_ (Dy_2.1_B_37_Si_9_) provide six and 6.3 electrons, respectively, and approximately six electrons are supplied from *RE* in *RE*_1–*x*_B_12_Si_3.3–*δ*_ (*RE* = Y, Gd–Lu) (0 ≤ *x* ≤ 0.5, *δ* ∼ 0.3) and *RE*_1–*x*_B_36_Si_9_C (*RE* = Y, Gd–Lu). The lattice constants and unit-cell volume of Mg_3_B_36_Si_9_C are *a* = 10.0793 Å, *c* = 16.372 Å, and *V* = 1440.4 Å^3^ (Ludwig *et al.* 2013 ▸), those of *RE*_1–*x*_B_12_Si_3.3–*δ*_ (*RE* = Y, Gd–Lu) (0 ≤ *x* ≤ 0.5, *δ* ∼ 0.3) are *a* = 10.046–10.095 Å, *c* = 16.298–16.467 Å, and *V* = 1429–1454 Å^3^ (Zhang *et al.* 2003 ▸) and those of *RE*_1–*x*_B_36_Si_9_C (*RE* = Y, Gd–Lu) are *a* = 10.000–10.096 Å, *c* = 16.225–16.454 Å, and *V* = 1405–1452 Å^3^ (Ludwig *et al.* 2013 ▸). Thus, it may be seen that the lattice constants of Na_3_MgB_37_Si_9_ are larger than those of related compounds and the unit-cell volume of Na_3_MgB_37_Si_9_ is approximately 2% larger than the maximum unit-cell volume of 1454 Å^3^ for the *RE*_1–*x*_B_12_Si_3.3–*δ*_ series with *RE* = Yb (Zhang *et al.* 2003 ▸). This increase in the lattice constants could be related to the occupancy of the Mg1 site, which is not found in other compounds.

Table 2 ▸ compares the inter­atomic distances for Na_3_MgB_37_Si_9_, Dy_2.1_B_37_Si_9_ and Mg_3_B_36_Si_9_C. The average B—B distances of B_12_ icosa­hedra, B2—B2 distances between clusters, and Si2—B4 distances for Na_3_MgB_37_Si_9_ are longer than those of other compounds. However, only the bond distance of Si3—Si3, in which Si3 only binds to Si, is specifically shorter. It is assumed that this bond became shorter because of an increase in the bond order from 1 because of a decrease in the number of electrons in the anti­bonding orbitals of the Si3—Si3 unit with a decrease in the electron count for the entire framework. Assuming that the main cause of the lattice expansion of Na_3_MgB_37_Si_9_ is a decrease in the bonding force between B—B and B—Si atoms because of electron deficiency in the bonding orbitals of the B_37_Si_9_ framework, the lattice constant can be reduced by increasing the Mg occupancy, which can be attained by increasing the Mg vapor pressure during the synthesis.

## Database survey

In space group *R* 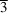 *m*, the framework structures of B_12_ icosa­hedral clusters containing {Si_8_} units similar to Na_3_MgB_37_Si_9_ have been reported for Mg_3_B_36_Si_9_C (Ludwig *et al.* 2013 ▸), *RE*_1–*x*_B_12_Si_3.3–*δ*_ (*RE* = Y, Gd–Lu) (0 ≤ *x* ≤ 0.5, *δ* ∼0.3) (Zhang *et al.* 2003 ▸) and *RE*_1–*x*_B_36_Si_9_C (*RE* = Y, Gd–Lu) (Ludwig *et al.* 2013 ▸).

## Synthesis and crystallization

Na metal pieces (purity 99.95%, Nippon Soda Co., Ltd.), crystalline B powder (99.9%, FUJIFILM Wako Pure Chemical Industries Co., Ltd.) and Si powder (99.999%, Kojundo Chemical Lab. Co., Ltd.) were weighed in a BN crucible (99.5%, Showa Denko K. K., outer diameter = 8.5 mm, inner diameter = 6.5 mm, depth = 18 mm), with a molar ratio of Na:B:Si = 5:4:3 (a total weight 280 mg) in a high-purity Ar-filled glove box (O_2_ < 1 ppm, H_2_O < 1 ppm). Then, 10 mg of B_2_O_3_ powder (90%, FUJIFILM Wako Pure Chemical Industries, Ltd.) were added to the crucible, which was stacked on another BN crucible containing 30 mg of Mg powder (99.9%, rare metallic), and these crucibles were encapsulated in a stainless steel container (SUS316, outer diameter = 12.7 mm, inner diameter = 10.75 mm, length 80 mm) with Ar gas. The container was heated at 1373 K for 24 h using an electric furnace. After cooling, the crucible was taken out from the reaction container, and any Na and NaSi remaining in the crucible were reacted and removed with 2-propanol and ethanol. Then, the sample was washed with pure water to remove water-soluble compounds such as sodium borate and alkoxide produced by the reaction of Na and alcohol to leave black plates of the title compound. An electron probe microanalyzer (EPMA; JEOL Ltd., JXA-8200) was used to analyze the composition of the obtained single crystal as Na 5.49 (8), Mg 2.37 (7), B 74.8 (7), Si 17.3 (4) atom %, which is nearly matched by Na_3_MgB_37_Si_9_ (Na 6.0, Mg 2.0, B 74.0, Si 18.0 atom %). Other elements such as O were not found.

## Refinement

Crystal data, data collection and structure refinement details are summarized in Table 3 ▸. The occupancy of the Mg1 site in the analysis of the initial model was 0.506 (10), whereas the occupancy of the B5 and Si1 sites was 0.519 (15) and 0.481, respectively. These occupancies were fixed at 0.5, and the composition formula was determined to be Na_3_MgB_37_Si_9_. The crystal structure was refined by considering (001) twinning, which reduced the *R*-value (all data) from 0.0651 to 0.0380.

## Supplementary Material

Crystal structure: contains datablock(s) I. DOI: 10.1107/S2056989022000494/hb8005sup1.cif

Structure factors: contains datablock(s) I. DOI: 10.1107/S2056989022000494/hb8005Isup2.hkl

CCDC reference: 2141726

Additional supporting information:  crystallographic information; 3D view; checkCIF report

## Figures and Tables

**Figure 2:**
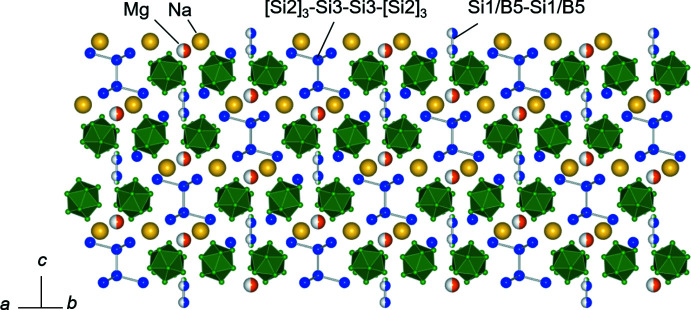
[110] projection of the crystal structure of Na_3_MgB_37_Si_9_.

**Table 1:** Selected geometric parameters (Å, °)

Na1—B2^i^	2.793 (2)	B2—B2^viii^	1.761 (5)
Na1—B1	2.811 (2)	B3—B5^i^	1.689 (7)
Na1—Si2^i^	2.8621 (4)	B3—Si1^i^	1.888 (4)
Na1—B4^i^	2.9605 (16)	B4—Si2	2.082 (3)
Mg1—B2^ii^	2.333 (3)	B5—B3^iii^	1.689 (7)
B1—B3^iii^	1.791 (3)	B5—Si1^ix^	1.96 (2)
B1—B2^iv^	1.798 (3)	B5—B5^ix^	2.47 (4)
B1—B1^v^	1.806 (4)	Si1—Si1^ix^	1.460 (10)
B1—B2^vi^	1.813 (3)	Si2—Si3^x^	2.3951 (9)
B1—B4^vii^	1.815 (3)	Si3—Si3^xi^	2.304 (3)
B1—Si2^i^	2.043 (2)		
			
Si3^xi^—Si3—Si2^x^	104.62 (4)	Si2^x^—Si3—Si2^xii^	113.86 (3)

Symmetry codes: (i) -x+{\script{2\over 3}}, -y+{\script{1\over 3}}, -z+{\script{1\over 3}}; (ii) -y, x-y, z; (iii) x-y-{\script{1\over 3}}, x-{\script{2\over 3}}, -z+{\script{1\over 3}}; (iv) x-y+{\script{2\over 3}}, -y+{\script{1\over 3}}, -z+{\script{1\over 3}}; (v) -x+{\script{2\over 3}}, -x+y+{\script{1\over 3}}, -z+{\script{1\over 3}}; (vi) -x+y, -x, z; (vii) -x+y+{\script{1\over 3}}, -x+{\script{2\over 3}}, z-{\script{1\over 3}}; (viii) -x+y, y, z; (ix) -x, -y, -z; (x) -x+{\script{1\over 3}}, -y+{\script{2\over 3}}, -z+{\script{2\over 3}}; (xi) -x, -y, -z+1; (xii) y-{\script{2\over 3}}, -x+y-{\script{1\over 3}}, -z+{\script{2\over 3}}.

**Table 2:** Cell parameters (Å), cell volumes (Å^3^) and selected bond lengths (Å) of Na_3_MgB_37_Si_9_, Dy_2.1_B_37_Si_9_^*a*^ and Mg_3_B_36_Si_9_C

	Na_3_MgB_37_Si_9_	Dy_2.1_B_37_Si_9_	Mg_3_B_36_Si_9_C
*a*	10.1630 (3)	10.078	10.079
*c*	16.5742 (6)	16.465	16.372
*V*	1482.54 (10)	1448.3	1440.4
B—B_av_ of B_12_ icosa­hedron	1.811	1.805	1.798
B2—B2	1.761 (5)	1.738	1.738
Si1—B3	1.887 (4)	1.877	1.851
Si1—B5/C	1.96 (2)	1.84	1.88
Si2—B1	2.043 (2)	2.032	2.035
Si2—B4	2.082 (3)	2.053	2.038
Si3—Si2	2.3951 (9)	2.366	2.362
Si3—Si3	2.304 (3)	2.343	2.341
Na1—B1	2.811 (2)	2.794	2.792
Na1—B2	2.793 (2)	2.751	2.729
Na1—B4	2.9604 (16)	2.934	2.934
Na1—Si2	2.8620 (4)	2.835	2.832
Mg1—B2	2.333 (3)		
Mg1—B4	2.568 (3)		
Mg1—Si3	2.403 (4)		

Notes: (*a*) Zhang *et al.* (2003 ▸); (*b*) Ludwig *et al.* (2013 ▸).

**Table 3:** Experimental details

Crystal data	
Chemical formula	Na_3_MgB_37_Si_9_
*M*_r_	746.06
Crystal system, space group	Trigonal, *R*\overline{3}*m*
Temperature (K)	298
*a*, *c* (Å)	10.1630 (3), 16.5742 (6)
*V* (Å^3^)	1482.54 (10)
*Z*	3
Radiation type	Mo *K*α
μ (mm^−1^)	0.72
Crystal size (mm)	0.20 × 0.16 × 0.02

Data collection	
Diffractometer	Burker, D8 QUEST
Absorption correction	Multi-scan (*SADABS*; Bruker, 2018 ▸)
*T*_min_, *T*_max_	0.911, 1.000
No. of measured, independent and observed [*I* > 2σ(*I*)] reflections	8352, 562, 540
*R*_int_	0.032
(sin θ/λ)_max_ (Å^−1^)	0.703

Refinement	
*R*[*F* ^2^ > 2σ(*F* ^2^)], *wR*(*F* ^2^), *S*	0.035, 0.076, 1.31
No. of reflections	562
No. of parameters	57
Δρ_max_, Δρ_min_ (e Å^−3^)	0.58, −0.53

Computer programs: *APEX3* and *SAINT* (Bruker, 2018 ▸), *SHELXT2014/5* (Sheldrick, 2015*a* ▸), *SHELXL2014/7* (Sheldrick, 2015*b* ▸), *VESTA* (Momma & Izumi, 2011 ▸) and *publCIF* (Westrip, 2010 ▸).

## supplementary crystallographic information

### Crystal data

**Table:**

Na_3_MgB_37_Si_9_	*D*_x_ = 2.507 Mg m^−^^3^
*M**_r_* = 746.06	Mo *K*α radiation, λ = 0.71073 Å
Trigonal, *R*3*m*	Cell parameters from 6032 reflections
*a* = 10.1630 (3) Å	θ = 3.7–41.2°
*c* = 16.5742 (6) Å	µ = 0.72 mm^−^^1^
*V* = 1482.54 (10) Å^3^	*T* = 298 K
*Z* = 3	Plate, black
*F*(000) = 1068	0.20 × 0.16 × 0.02 mm

### Data collection

**Table:**

Burker, D8 QUEST diffractometer	540 reflections with *I* > 2σ(*I*)
Detector resolution: 10 pixels mm^-1^	*R*_int_ = 0.032
ω scans	θ_max_ = 30.0°, θ_min_ = 2.6°
Absorption correction: multi-scan (SADABS; Bruker, 2018)	*h* = −14→14
*T*_min_ = 0.911, *T*_max_ = 1.000	*k* = −14→14
8352 measured reflections	*l* = −23→22
562 independent reflections	

### Refinement

**Table:**

Refinement on *F*^2^	0 restraints
Least-squares matrix: full	*w* = 1/[σ^2^(*F*_o_^2^) + 11.3797*P*] where *P* = (*F*_o_^2^ + 2*F*_c_^2^)/3
*R*[*F*^2^ > 2σ(*F*^2^)] = 0.035	(Δ/σ)_max_ < 0.001
*wR*(*F*^2^) = 0.076	Δρ_max_ = 0.58 e Å^−^^3^
*S* = 1.31	Δρ_min_ = −0.53 e Å^−^^3^
562 reflections	Extinction correction: SHELXL2014/7 (Sheldrick 2015), Fc^*^=kFc[1+0.001xFc^2^λ^3^/sin(2θ)]^-1/4^
57 parameters	Extinction coefficient: 0.0030 (6)

### Special details

**Table:**

Geometry. All esds (except the esd in the dihedral angle between two l.s. planes) are estimated using the full covariance matrix. The cell esds are taken into account individually in the estimation of esds in distances, angles and torsion angles; correlations between esds in cell parameters are only used when they are defined by crystal symmetry. An approximate (isotropic) treatment of cell esds is used for estimating esds involving l.s. planes.
Refinement. Refined as a 2-component inversion twin.

### Fractional atomic coordinates and isotropic or equivalent isotropic displacement parameters (Å^2^)

**Table:**

	*x*	*y*	*z*	*U*_iso_*/*U*_eq_	Occ. (<1)
Na1	0.5000	0.0000	0.0000	0.0179 (5)	
Mg1	0.0000	0.0000	0.2855 (2)	0.0074 (7)	0.5
B1	0.3002 (3)	0.0065 (2)	0.11511 (13)	0.0064 (4)	
B2	0.0027 (3)	0.1787 (3)	0.19610 (13)	0.0072 (4)	
B3	0.7591 (2)	0.2409 (2)	0.2315 (2)	0.0116 (7)	
B4	0.47839 (19)	0.52161 (19)	0.39743 (19)	0.0079 (6)	
B5	0.0000	0.0000	0.0744 (12)	0.026 (5)	0.5
Si1	0.0000	0.0000	0.0441 (3)	0.0103 (9)	0.5
Si2	0.46499 (5)	0.53501 (5)	0.27264 (5)	0.0056 (2)	
Si3	0.0000	0.0000	0.43049 (10)	0.0120 (3)	

### Atomic displacement parameters (Å^2^)

**Table:**

	*U*^11^	*U*^22^	*U*^33^	*U*^12^	*U*^13^	*U*^23^
Na1	0.0137 (7)	0.0265 (11)	0.0178 (8)	0.0132 (6)	0.0027 (4)	0.0054 (8)
Mg1	0.0060 (9)	0.0060 (9)	0.0102 (15)	0.0030 (5)	0.000	0.000
B1	0.0064 (9)	0.0041 (9)	0.0080 (8)	0.0020 (8)	−0.0004 (7)	0.0001 (8)
B2	0.0054 (9)	0.0053 (9)	0.0102 (9)	0.0022 (8)	−0.0005 (8)	−0.0009 (8)
B3	0.0087 (10)	0.0087 (10)	0.0116 (13)	0.0001 (12)	0.0035 (7)	−0.0035 (7)
B4	0.0050 (9)	0.0050 (9)	0.0116 (13)	0.0009 (11)	−0.0004 (6)	0.0004 (6)
B5	0.033 (8)	0.033 (8)	0.012 (9)	0.017 (4)	0.000	0.000
Si1	0.0061 (11)	0.0061 (11)	0.019 (3)	0.0031 (5)	0.000	0.000
Si2	0.0044 (3)	0.0044 (3)	0.0073 (4)	0.0015 (3)	0.00040 (14)	−0.00040 (14)
Si3	0.0055 (4)	0.0055 (4)	0.0249 (8)	0.0028 (2)	0.000	0.000

### Geometric parameters (Å, º)

**Table:**

Na1—B2^i^	2.793 (2)	B3—Si1^i^	1.888 (4)
Na1—B2^ii^	2.793 (2)	B3—B5^xxx^	3.343 (19)
Na1—B2^iii^	2.793 (2)	B3—Na1^xxx^	4.123 (3)
Na1—B2^iv^	2.793 (2)	B3—Na1^xxxi^	4.123 (3)
Na1—B1^v^	2.811 (2)	B3—Na1^xxxii^	4.605 (3)
Na1—B1^vi^	2.811 (2)	B4—B3^xxviii^	1.799 (5)
Na1—B1^vii^	2.811 (2)	B4—B1^xxxiii^	1.815 (3)
Na1—B1	2.811 (2)	B4—B1^xxxiv^	1.815 (3)
Na1—Si2^viii^	2.8620 (4)	B4—B2^xxxv^	1.824 (4)
Na1—Si2^ix^	2.8620 (4)	B4—B2^xv^	1.824 (4)
Na1—Si2^i^	2.8621 (4)	B4—Si2	2.082 (3)
Na1—Si2^ii^	2.8621 (4)	B4—Mg1^xv^	2.568 (3)
Na1—B4^viii^	2.9604 (16)	B4—Na1^xxiv^	2.9605 (16)
Na1—B4^ix^	2.9604 (16)	B4—Na1^xxxiii^	2.9605 (16)
Na1—B4^i^	2.9605 (16)	B4—B5^xxx^	3.319 (3)
Na1—B4^ii^	2.9605 (16)	B4—B5^i^	4.031 (12)
Na1—Mg1^i^	3.0389 (9)	B4—B5^xv^	4.117 (16)
Na1—Mg1^ii^	3.0389 (9)	B5—Si1	0.503 (18)
Mg1—B2^x^	2.333 (3)	B5—B3^xviii^	1.689 (7)
Mg1—B2^xi^	2.333 (3)	B5—B3^xxxvi^	1.689 (7)
Mg1—B2^xii^	2.333 (3)	B5—B3^i^	1.689 (7)
Mg1—B2^xiii^	2.333 (3)	B5—Si1^xxi^	1.96 (2)
Mg1—B2^xiv^	2.333 (3)	B5—B5^xxi^	2.47 (4)
Mg1—B2	2.333 (3)	B5—B2^xiii^	2.705 (16)
Mg1—Si3	2.403 (4)	B5—B2^xiv^	2.705 (16)
Mg1—B4^xv^	2.568 (3)	B5—B2^xi^	2.705 (16)
Mg1—B4^xvi^	2.568 (3)	B5—B2^xii^	2.705 (16)
Mg1—B4^xvii^	2.568 (3)	B5—B2^x^	2.705 (16)
Mg1—Si2^xv^	2.933 (2)	Si1—Si1^xxi^	1.460 (10)
Mg1—Si2^xvii^	2.933 (2)	Si1—B3^xviii^	1.887 (4)
B1—B3^xviii^	1.791 (3)	Si1—B3^xxxvi^	1.887 (4)
B1—B2^iv^	1.798 (3)	Si1—B3^i^	1.888 (4)
B1—B1^xix^	1.806 (4)	Si1—B5^xxi^	1.96 (2)
B1—B2^xiv^	1.813 (3)	Si1—Na1^xiv^	5.1337 (7)
B1—B4^ix^	1.815 (3)	Si1—Na1^xxii^	5.1337 (7)
B1—Si2^i^	2.043 (2)	Si1—Na1^xxxvii^	5.1337 (7)
B1—B5	3.093 (5)	Si1—Na1^xxxviii^	5.1337 (7)
B1—Na1^xx^	3.954 (2)	Si1—Na1^x^	5.1337 (7)
B1—B5^i^	4.268 (12)	Si2—B1^i^	2.043 (2)
B1—B5^xxi^	4.356 (15)	Si2—B1^xxix^	2.043 (2)
B1—Na1^xxii^	4.768 (2)	Si2—Si3^xv^	2.3951 (9)
B2—B2^xiii^	1.761 (5)	Si2—Na1^xxxiii^	2.8621 (4)
B2—B1^xxiii^	1.798 (3)	Si2—Na1^xxiv^	2.8621 (4)
B2—B1^x^	1.813 (3)	Si2—Mg1^xv^	2.933 (2)
B2—B3^i^	1.816 (4)	Si2—B5^i^	3.5572 (16)
B2—B4^xv^	1.824 (4)	Si2—B5^xxx^	4.197 (11)
B2—B2^xi^	1.843 (5)	Si2—Na1^xxii^	4.5605 (8)
B2—B5	2.705 (16)	Si2—Na1^xxxix^	5.3470 (8)
B2—Na1^xxiv^	2.793 (2)	Si3—Si3^xl^	2.304 (3)
B2—Na1^xxv^	4.143 (2)	Si3—Si2^xv^	2.3951 (9)
B2—B5^xxvi^	4.537 (5)	Si3—Si2^xvi^	2.3952 (9)
B2—Na1^x^	4.617 (2)	Si3—Si2^xvii^	2.3952 (9)
B3—B5^i^	1.689 (7)	Si3—Na1^xx^	3.3466 (8)
B3—B1^xxvii^	1.791 (3)	Si3—Na1^xxv^	3.3467 (8)
B3—B1^iv^	1.791 (3)	Si3—Na1^xxiv^	3.3467 (8)
B3—B4^xxviii^	1.799 (5)	Si3—Na1^xli^	4.8918 (14)
B3—B2^i^	1.816 (4)	Si3—Na1^xlii^	4.8918 (14)
B3—B2^xxix^	1.816 (4)	Si3—Na1^xliii^	4.8918 (14)
			
B2^i^—Na1—B2^ii^	180.00 (5)	B1^iv^—B3—B2^xxix^	60.33 (12)
B2^i^—Na1—B2^iii^	143.25 (9)	B4^xxviii^—B3—B2^xxix^	109.8 (2)
B2^ii^—Na1—B2^iii^	36.75 (9)	B2^i^—B3—B2^xxix^	60.99 (18)
B2^i^—Na1—B2^iv^	36.75 (9)	B5^i^—B3—Si1^i^	14.9 (6)
B2^ii^—Na1—B2^iv^	143.25 (9)	B1^xxvii^—B3—Si1^i^	123.43 (12)
B2^iii^—Na1—B2^iv^	180.00 (11)	B1^iv^—B3—Si1^i^	123.43 (12)
B2^i^—Na1—B1^v^	109.34 (7)	B4^xxviii^—B3—Si1^i^	129.2 (2)
B2^ii^—Na1—B1^v^	70.66 (7)	B2^i^—B3—Si1^i^	113.5 (2)
B2^iii^—Na1—B1^v^	37.43 (6)	B2^xxix^—B3—Si1^i^	113.5 (2)
B2^iv^—Na1—B1^v^	142.57 (6)	B5^i^—B3—B5^xxx^	45.3 (8)
B2^i^—Na1—B1^vi^	37.43 (6)	B1^xxvii^—B3—B5^xxx^	112.55 (16)
B2^ii^—Na1—B1^vi^	142.57 (6)	B1^iv^—B3—B5^xxx^	112.55 (16)
B2^iii^—Na1—B1^vi^	109.34 (7)	B4^xxviii^—B3—B5^xxx^	98.8 (3)
B2^iv^—Na1—B1^vi^	70.66 (7)	B2^i^—B3—B5^xxx^	136.96 (19)
B1^v^—Na1—B1^vi^	85.53 (9)	B2^xxix^—B3—B5^xxx^	136.96 (19)
B2^i^—Na1—B1^vii^	142.57 (6)	Si1^i^—B3—B5^xxx^	30.4 (3)
B2^ii^—Na1—B1^vii^	37.43 (6)	B5^i^—B3—Na1^xxx^	122.5 (5)
B2^iii^—Na1—B1^vii^	70.66 (7)	B1^xxvii^—B3—Na1^xxx^	99.85 (16)
B2^iv^—Na1—B1^vii^	109.34 (7)	B1^iv^—B3—Na1^xxx^	33.59 (11)
B1^v^—Na1—B1^vii^	94.47 (9)	B4^xxviii^—B3—Na1^xxx^	39.37 (5)
B1^vi^—Na1—B1^vii^	180.00 (6)	B2^i^—B3—Na1^xxx^	133.94 (17)
B2^i^—Na1—B1	70.66 (7)	B2^xxix^—B3—Na1^xxx^	93.92 (11)
B2^ii^—Na1—B1	109.34 (7)	Si1^i^—B3—Na1^xxx^	111.86 (14)
B2^iii^—Na1—B1	142.57 (6)	B5^xxx^—B3—Na1^xxx^	88.28 (16)
B2^iv^—Na1—B1	37.43 (6)	B5^i^—B3—Na1^xxxi^	122.5 (5)
B1^v^—Na1—B1	180.0	B1^xxvii^—B3—Na1^xxxi^	33.59 (11)
B1^vi^—Na1—B1	94.47 (9)	B1^iv^—B3—Na1^xxxi^	99.85 (16)
B1^vii^—Na1—B1	85.53 (9)	B4^xxviii^—B3—Na1^xxxi^	39.37 (5)
B2^i^—Na1—Si2^viii^	78.17 (5)	B2^i^—B3—Na1^xxxi^	93.92 (11)
B2^ii^—Na1—Si2^viii^	101.83 (5)	B2^xxix^—B3—Na1^xxxi^	133.94 (17)
B2^iii^—Na1—Si2^viii^	76.28 (5)	Si1^i^—B3—Na1^xxxi^	111.86 (14)
B2^iv^—Na1—Si2^viii^	103.72 (5)	B5^xxx^—B3—Na1^xxxi^	88.28 (16)
B1^v^—Na1—Si2^viii^	73.21 (5)	Na1^xxx^—B3—Na1^xxxi^	76.09 (7)
B1^vi^—Na1—Si2^viii^	42.21 (5)	B5^i^—B3—Na1	101.0 (6)
B1^vii^—Na1—Si2^viii^	137.79 (5)	B1^xxvii^—B3—Na1	57.83 (11)
B1—Na1—Si2^viii^	106.79 (5)	B1^iv^—B3—Na1	111.50 (15)
B2^i^—Na1—Si2^ix^	101.83 (5)	B4^xxviii^—B3—Na1	108.78 (14)
B2^ii^—Na1—Si2^ix^	78.17 (5)	B2^i^—B3—Na1	3.02 (8)
B2^iii^—Na1—Si2^ix^	103.72 (5)	B2^xxix^—B3—Na1	63.98 (11)
B2^iv^—Na1—Si2^ix^	76.28 (5)	Si1^i^—B3—Na1	113.12 (15)
B1^v^—Na1—Si2^ix^	106.79 (5)	B5^xxx^—B3—Na1	135.32 (13)
B1^vi^—Na1—Si2^ix^	137.79 (5)	Na1^xxx^—B3—Na1	134.78 (8)
B1^vii^—Na1—Si2^ix^	42.21 (5)	Na1^xxxi^—B3—Na1	91.40 (4)
B1—Na1—Si2^ix^	73.21 (5)	B5^i^—B3—Na1^xxxii^	101.0 (6)
Si2^viii^—Na1—Si2^ix^	180.00 (3)	B1^xxvii^—B3—Na1^xxxii^	111.50 (15)
B2^i^—Na1—Si2^i^	103.71 (5)	B1^iv^—B3—Na1^xxxii^	57.83 (11)
B2^ii^—Na1—Si2^i^	76.29 (5)	B4^xxviii^—B3—Na1^xxxii^	108.78 (14)
B2^iii^—Na1—Si2^i^	101.83 (5)	B2^i^—B3—Na1^xxxii^	63.98 (11)
B2^iv^—Na1—Si2^i^	78.17 (5)	B2^xxix^—B3—Na1^xxxii^	3.02 (8)
B1^v^—Na1—Si2^i^	137.79 (5)	Si1^i^—B3—Na1^xxxii^	113.12 (15)
B1^vi^—Na1—Si2^i^	106.80 (5)	B5^xxx^—B3—Na1^xxxii^	135.32 (13)
B1^vii^—Na1—Si2^i^	73.20 (5)	Na1^xxx^—B3—Na1^xxxii^	91.40 (4)
B1—Na1—Si2^i^	42.21 (5)	Na1^xxxi^—B3—Na1^xxxii^	134.78 (8)
Si2^viii^—Na1—Si2^i^	89.06 (3)	Na1—B3—Na1^xxxii^	66.97 (5)
Si2^ix^—Na1—Si2^i^	90.94 (3)	B3^xxviii^—B4—B1^xxxiii^	59.41 (12)
B2^i^—Na1—Si2^ii^	76.29 (5)	B3^xxviii^—B4—B1^xxxiv^	59.41 (12)
B2^ii^—Na1—Si2^ii^	103.71 (5)	B1^xxxiii^—B4—B1^xxxiv^	107.0 (2)
B2^iii^—Na1—Si2^ii^	78.17 (5)	B3^xxviii^—B4—B2^xxxv^	106.60 (19)
B2^iv^—Na1—Si2^ii^	101.83 (5)	B1^xxxiii^—B4—B2^xxxv^	59.22 (12)
B1^v^—Na1—Si2^ii^	42.21 (5)	B1^xxxiv^—B4—B2^xxxv^	107.49 (19)
B1^vi^—Na1—Si2^ii^	73.20 (5)	B3^xxviii^—B4—B2^xv^	106.60 (19)
B1^vii^—Na1—Si2^ii^	106.80 (5)	B1^xxxiii^—B4—B2^xv^	107.49 (19)
B1—Na1—Si2^ii^	137.79 (5)	B1^xxxiv^—B4—B2^xv^	59.22 (12)
Si2^viii^—Na1—Si2^ii^	90.94 (3)	B2^xxxv^—B4—B2^xv^	60.69 (17)
Si2^ix^—Na1—Si2^ii^	89.06 (3)	B3^xxviii^—B4—Si2	116.8 (2)
Si2^i^—Na1—Si2^ii^	180.00 (3)	B1^xxxiii^—B4—Si2	120.31 (12)
B2^i^—Na1—B4^viii^	109.24 (8)	B1^xxxiv^—B4—Si2	120.31 (12)
B2^ii^—Na1—B4^viii^	70.76 (8)	B2^xxxv^—B4—Si2	126.71 (16)
B2^iii^—Na1—B4^viii^	36.82 (8)	B2^xv^—B4—Si2	126.71 (16)
B2^iv^—Na1—B4^viii^	143.18 (8)	B3^xxviii^—B4—Mg1^xv^	165.7 (2)
B1^v^—Na1—B4^viii^	36.55 (7)	B1^xxxiii^—B4—Mg1^xv^	114.65 (14)
B1^vi^—Na1—B4^viii^	72.94 (8)	B1^xxxiv^—B4—Mg1^xv^	114.65 (14)
B1^vii^—Na1—B4^viii^	107.06 (8)	B2^xxxv^—B4—Mg1^xv^	61.46 (13)
B1—Na1—B4^viii^	143.45 (7)	B2^xv^—B4—Mg1^xv^	61.46 (13)
Si2^viii^—Na1—B4^viii^	41.86 (6)	Si2—B4—Mg1^xv^	77.46 (13)
Si2^ix^—Na1—B4^viii^	138.14 (6)	B3^xxviii^—B4—Na1^xxiv^	117.95 (7)
Si2^i^—Na1—B4^viii^	107.63 (6)	B1^xxxiii^—B4—Na1^xxiv^	173.12 (16)
Si2^ii^—Na1—B4^viii^	72.37 (6)	B1^xxxiv^—B4—Na1^xxiv^	67.24 (8)
B2^i^—Na1—B4^ix^	70.76 (8)	B2^xxxv^—B4—Na1^xxiv^	118.03 (15)
B2^ii^—Na1—B4^ix^	109.24 (8)	B2^xv^—B4—Na1^xxiv^	66.59 (8)
B2^iii^—Na1—B4^ix^	143.18 (8)	Si2—B4—Na1^xxiv^	66.54 (6)
B2^iv^—Na1—B4^ix^	36.82 (8)	Mg1^xv^—B4—Na1^xxiv^	66.25 (7)
B1^v^—Na1—B4^ix^	143.45 (7)	B3^xxviii^—B4—Na1^xxxiii^	117.95 (7)
B1^vi^—Na1—B4^ix^	107.06 (8)	B1^xxxiii^—B4—Na1^xxxiii^	67.24 (8)
B1^vii^—Na1—B4^ix^	72.94 (8)	B1^xxxiv^—B4—Na1^xxxiii^	173.12 (16)
B1—Na1—B4^ix^	36.55 (7)	B2^xxxv^—B4—Na1^xxxiii^	66.59 (8)
Si2^viii^—Na1—B4^ix^	138.14 (6)	B2^xv^—B4—Na1^xxxiii^	118.03 (15)
Si2^ix^—Na1—B4^ix^	41.86 (6)	Si2—B4—Na1^xxxiii^	66.54 (6)
Si2^i^—Na1—B4^ix^	72.37 (6)	Mg1^xv^—B4—Na1^xxxiii^	66.25 (7)
Si2^ii^—Na1—B4^ix^	107.63 (6)	Na1^xxiv^—B4—Na1^xxxiii^	118.24 (11)
B4^viii^—Na1—B4^ix^	180.00 (18)	B3^xxviii^—B4—B5^xxx^	17.4 (4)
B2^i^—Na1—B4^i^	143.17 (8)	B1^xxxiii^—B4—B5^xxx^	66.8 (2)
B2^ii^—Na1—B4^i^	36.83 (8)	B1^xxxiv^—B4—B5^xxx^	66.8 (2)
B2^iii^—Na1—B4^i^	70.76 (8)	B2^xxxv^—B4—B5^xxx^	121.1 (3)
B2^iv^—Na1—B4^i^	109.24 (8)	B2^xv^—B4—B5^xxx^	121.1 (3)
B1^v^—Na1—B4^i^	107.06 (8)	Si2—B4—B5^xxx^	99.4 (4)
B1^vi^—Na1—B4^i^	143.45 (7)	Mg1^xv^—B4—B5^xxx^	176.9 (4)
B1^vii^—Na1—B4^i^	36.55 (7)	Na1^xxiv^—B4—B5^xxx^	112.64 (15)
B1—Na1—B4^i^	72.94 (8)	Na1^xxxiii^—B4—B5^xxx^	112.64 (15)
Si2^viii^—Na1—B4^i^	107.63 (6)	B3^xxviii^—B4—B5^i^	55.0 (3)
Si2^ix^—Na1—B4^i^	72.37 (6)	B1^xxxiii^—B4—B5^i^	87.8 (2)
Si2^i^—Na1—B4^i^	41.85 (6)	B1^xxxiv^—B4—B5^i^	87.8 (2)
Si2^ii^—Na1—B4^i^	138.15 (6)	B2^xxxv^—B4—B5^i^	146.20 (15)
B4^viii^—Na1—B4^i^	96.65 (12)	B2^xv^—B4—B5^i^	146.20 (15)
B4^ix^—Na1—B4^i^	83.35 (12)	Si2—B4—B5^i^	61.8 (3)
B2^i^—Na1—B4^ii^	36.83 (8)	Mg1^xv^—B4—B5^i^	139.3 (3)
B2^ii^—Na1—B4^ii^	143.17 (8)	Na1^xxiv^—B4—B5^i^	95.59 (14)
B2^iii^—Na1—B4^ii^	109.24 (8)	Na1^xxxiii^—B4—B5^i^	95.59 (14)
B2^iv^—Na1—B4^ii^	70.76 (8)	B5^xxx^—B4—B5^i^	37.6 (6)
B1^v^—Na1—B4^ii^	72.94 (8)	B3^xxviii^—B4—B5^xv^	108.0 (2)
B1^vi^—Na1—B4^ii^	36.55 (7)	B1^xxxiii^—B4—B5^xv^	82.20 (16)
B1^vii^—Na1—B4^ii^	143.45 (7)	B1^xxxiv^—B4—B5^xv^	82.20 (16)
B1—Na1—B4^ii^	107.06 (8)	B2^xxxv^—B4—B5^xv^	30.37 (9)
Si2^viii^—Na1—B4^ii^	72.37 (6)	B2^xv^—B4—B5^xv^	30.37 (9)
Si2^ix^—Na1—B4^ii^	107.63 (6)	Si2—B4—B5^xv^	135.2 (2)
Si2^i^—Na1—B4^ii^	138.15 (6)	Mg1^xv^—B4—B5^xv^	57.7 (2)
Si2^ii^—Na1—B4^ii^	41.85 (6)	Na1^xxiv^—B4—B5^xv^	93.09 (12)
B4^viii^—Na1—B4^ii^	83.35 (12)	Na1^xxxiii^—B4—B5^xv^	93.09 (12)
B4^ix^—Na1—B4^ii^	96.65 (12)	B5^xxx^—B4—B5^xv^	125.4 (2)
B4^i^—Na1—B4^ii^	180.00 (13)	B5^i^—B4—B5^xv^	163.0 (4)
B2^i^—Na1—Mg1^i^	46.93 (7)	Si1—B5—B3^xviii^	105.6 (7)
B2^ii^—Na1—Mg1^i^	133.07 (7)	Si1—B5—B3^xxxvi^	105.6 (7)
B2^iii^—Na1—Mg1^i^	133.07 (7)	B3^xviii^—B5—B3^xxxvi^	113.0 (6)
B2^iv^—Na1—Mg1^i^	46.93 (7)	Si1—B5—B3^i^	105.6 (7)
B1^v^—Na1—Mg1^i^	101.34 (6)	B3^xviii^—B5—B3^i^	113.0 (6)
B1^vi^—Na1—Mg1^i^	78.66 (6)	B3^xxxvi^—B5—B3^i^	113.0 (6)
B1^vii^—Na1—Mg1^i^	101.34 (6)	Si1—B5—Si1^xxi^	0.0
B1—Na1—Mg1^i^	78.65 (6)	B3^xviii^—B5—Si1^xxi^	105.6 (7)
Si2^viii^—Na1—Mg1^i^	120.47 (4)	B3^xxxvi^—B5—Si1^xxi^	105.6 (7)
Si2^ix^—Na1—Mg1^i^	59.53 (4)	B3^i^—B5—Si1^xxi^	105.6 (7)
Si2^i^—Na1—Mg1^i^	120.47 (4)	Si1—B5—B5^xxi^	0.000 (1)
Si2^ii^—Na1—Mg1^i^	59.53 (4)	B3^xviii^—B5—B5^xxi^	105.6 (7)
B4^viii^—Na1—Mg1^i^	129.34 (6)	B3^xxxvi^—B5—B5^xxi^	105.6 (7)
B4^ix^—Na1—Mg1^i^	50.66 (6)	B3^i^—B5—B5^xxi^	105.6 (7)
B4^i^—Na1—Mg1^i^	129.34 (6)	Si1^xxi^—B5—B5^xxi^	0.0
B4^ii^—Na1—Mg1^i^	50.66 (6)	Si1—B5—B2	138.2 (3)
B2^i^—Na1—Mg1^ii^	133.07 (7)	B3^xviii^—B5—B2	77.9 (5)
B2^ii^—Na1—Mg1^ii^	46.93 (7)	B3^xxxvi^—B5—B2	111.0 (9)
B2^iii^—Na1—Mg1^ii^	46.93 (7)	B3^i^—B5—B2	41.2 (4)
B2^iv^—Na1—Mg1^ii^	133.07 (7)	Si1^xxi^—B5—B2	138.2 (3)
B1^v^—Na1—Mg1^ii^	78.66 (6)	B5^xxi^—B5—B2	138.2 (3)
B1^vi^—Na1—Mg1^ii^	101.34 (6)	Si1—B5—B2^xiii^	138.2 (3)
B1^vii^—Na1—Mg1^ii^	78.66 (6)	B3^xviii^—B5—B2^xiii^	41.2 (4)
B1—Na1—Mg1^ii^	101.35 (6)	B3^xxxvi^—B5—B2^xiii^	111.1 (9)
Si2^viii^—Na1—Mg1^ii^	59.53 (4)	B3^i^—B5—B2^xiii^	77.9 (5)
Si2^ix^—Na1—Mg1^ii^	120.47 (4)	Si1^xxi^—B5—B2^xiii^	138.2 (3)
Si2^i^—Na1—Mg1^ii^	59.53 (4)	B5^xxi^—B5—B2^xiii^	138.2 (3)
Si2^ii^—Na1—Mg1^ii^	120.47 (4)	B2—B5—B2^xiii^	38.0 (2)
B4^viii^—Na1—Mg1^ii^	50.66 (6)	Si1—B5—B2^xiv^	138.2 (3)
B4^ix^—Na1—Mg1^ii^	129.34 (6)	B3^xviii^—B5—B2^xiv^	41.2 (4)
B4^i^—Na1—Mg1^ii^	50.66 (6)	B3^xxxvi^—B5—B2^xiv^	77.9 (5)
B4^ii^—Na1—Mg1^ii^	129.34 (6)	B3^i^—B5—B2^xiv^	111.1 (9)
Mg1^i^—Na1—Mg1^ii^	180.00 (13)	Si1^xxi^—B5—B2^xiv^	138.2 (3)
B2^x^—Mg1—B2^xi^	44.34 (12)	B5^xxi^—B5—B2^xiv^	138.2 (3)
B2^x^—Mg1—B2^xii^	46.53 (12)	B2—B5—B2^xiv^	70.5 (5)
B2^xi^—Mg1—B2^xii^	83.96 (13)	B2^xiii^—B5—B2^xiv^	39.8 (3)
B2^x^—Mg1—B2^xiii^	101.12 (17)	Si1—B5—B2^xi^	138.2 (3)
B2^xi^—Mg1—B2^xiii^	83.96 (13)	B3^xviii^—B5—B2^xi^	111.0 (9)
B2^xii^—Mg1—B2^xiii^	83.96 (13)	B3^xxxvi^—B5—B2^xi^	77.9 (5)
B2^x^—Mg1—B2^xiv^	83.96 (13)	B3^i^—B5—B2^xi^	41.2 (4)
B2^xi^—Mg1—B2^xiv^	101.12 (17)	Si1^xxi^—B5—B2^xi^	138.2 (3)
B2^xii^—Mg1—B2^xiv^	44.34 (12)	B5^xxi^—B5—B2^xi^	138.2 (3)
B2^xiii^—Mg1—B2^xiv^	46.53 (12)	B2—B5—B2^xi^	39.8 (3)
B2^x^—Mg1—B2	83.96 (13)	B2^xiii^—B5—B2^xi^	70.5 (5)
B2^xi^—Mg1—B2	46.52 (12)	B2^xiv^—B5—B2^xi^	83.5 (6)
B2^xii^—Mg1—B2	101.12 (17)	Si1—B5—B2^xii^	138.2 (3)
B2^xiii^—Mg1—B2	44.34 (12)	B3^xviii^—B5—B2^xii^	77.9 (5)
B2^xiv^—Mg1—B2	83.96 (13)	B3^xxxvi^—B5—B2^xii^	41.2 (4)
B2^x^—Mg1—Si3	129.43 (9)	B3^i^—B5—B2^xii^	111.1 (9)
B2^xi^—Mg1—Si3	129.43 (9)	Si1^xxi^—B5—B2^xii^	138.2 (3)
B2^xii^—Mg1—Si3	129.43 (9)	B5^xxi^—B5—B2^xii^	138.2 (3)
B2^xiii^—Mg1—Si3	129.43 (9)	B2—B5—B2^xii^	83.5 (6)
B2^xiv^—Mg1—Si3	129.43 (9)	B2^xiii^—B5—B2^xii^	70.5 (5)
B2—Mg1—Si3	129.43 (9)	B2^xiv^—B5—B2^xii^	38.0 (2)
B2^x^—Mg1—B4^xv^	85.59 (9)	B2^xi^—B5—B2^xii^	70.5 (5)
B2^xi^—Mg1—B4^xv^	43.37 (9)	Si1—B5—B2^x^	138.2 (3)
B2^xii^—Mg1—B4^xv^	127.11 (15)	B3^xviii^—B5—B2^x^	111.1 (9)
B2^xiii^—Mg1—B4^xv^	85.59 (9)	B3^xxxvi^—B5—B2^x^	41.2 (4)
B2^xiv^—Mg1—B4^xv^	127.11 (15)	B3^i^—B5—B2^x^	77.9 (5)
B2—Mg1—B4^xv^	43.37 (9)	Si1^xxi^—B5—B2^x^	138.2 (3)
Si3—Mg1—B4^xv^	96.04 (11)	B5^xxi^—B5—B2^x^	138.2 (3)
B2^x^—Mg1—B4^xvi^	43.37 (9)	B2—B5—B2^x^	70.5 (5)
B2^xi^—Mg1—B4^xvi^	85.59 (9)	B2^xiii^—B5—B2^x^	83.5 (6)
B2^xii^—Mg1—B4^xvi^	43.37 (9)	B2^xiv^—B5—B2^x^	70.5 (5)
B2^xiii^—Mg1—B4^xvi^	127.11 (15)	B2^xi^—B5—B2^x^	38.0 (2)
B2^xiv^—Mg1—B4^xvi^	85.59 (9)	B2^xii^—B5—B2^x^	39.8 (3)
B2—Mg1—B4^xvi^	127.11 (15)	B5—Si1—Si1^xxi^	180.0
Si3—Mg1—B4^xvi^	96.04 (11)	B5—Si1—B3^xviii^	59.53 (17)
B4^xv^—Mg1—B4^xvi^	118.91 (4)	Si1^xxi^—Si1—B3^xviii^	120.47 (17)
B2^x^—Mg1—B4^xvii^	127.11 (15)	B5—Si1—B3^xxxvi^	59.53 (17)
B2^xi^—Mg1—B4^xvii^	127.11 (15)	Si1^xxi^—Si1—B3^xxxvi^	120.47 (17)
B2^xii^—Mg1—B4^xvii^	85.59 (9)	B3^xviii^—Si1—B3^xxxvi^	96.6 (2)
B2^xiii^—Mg1—B4^xvii^	43.37 (9)	B5—Si1—B3^i^	59.53 (17)
B2^xiv^—Mg1—B4^xvii^	43.37 (9)	Si1^xxi^—Si1—B3^i^	120.47 (17)
B2—Mg1—B4^xvii^	85.59 (9)	B3^xviii^—Si1—B3^i^	96.6 (2)
Si3—Mg1—B4^xvii^	96.04 (11)	B3^xxxvi^—Si1—B3^i^	96.6 (2)
B4^xv^—Mg1—B4^xvii^	118.91 (4)	B5—Si1—B5^xxi^	180.0
B4^xvi^—Mg1—B4^xvii^	118.91 (4)	Si1^xxi^—Si1—B5^xxi^	0.0
B2^x^—Mg1—Si2^xv^	112.42 (6)	B3^xviii^—Si1—B5^xxi^	120.47 (17)
B2^xi^—Mg1—Si2^xv^	82.24 (6)	B3^xxxvi^—Si1—B5^xxi^	120.47 (17)
B2^xii^—Mg1—Si2^xv^	157.19 (6)	B3^i^—Si1—B5^xxi^	120.47 (17)
B2^xiii^—Mg1—Si2^xv^	112.42 (6)	B5—Si1—Na1^xiv^	98.18 (5)
B2^xiv^—Mg1—Si2^xv^	157.19 (6)	Si1^xxi^—Si1—Na1^xiv^	81.82 (5)
B2—Mg1—Si2^xv^	82.24 (6)	B3^xviii^—Si1—Na1^xiv^	144.19 (12)
Si3—Mg1—Si2^xv^	52.19 (6)	B3^xxxvi^—Si1—Na1^xiv^	48.18 (10)
B4^xv^—Mg1—Si2^xv^	43.85 (8)	B3^i^—Si1—Na1^xiv^	94.136 (18)
B4^xvi^—Mg1—Si2^xv^	117.22 (8)	B5^xxi^—Si1—Na1^xiv^	81.82 (5)
B4^xvii^—Mg1—Si2^xv^	117.22 (8)	B5—Si1—Na1^xxii^	98.18 (5)
B2^x^—Mg1—Si2^xvii^	157.19 (6)	Si1^xxi^—Si1—Na1^xxii^	81.82 (5)
B2^xi^—Mg1—Si2^xvii^	157.19 (6)	B3^xviii^—Si1—Na1^xxii^	48.18 (10)
B2^xii^—Mg1—Si2^xvii^	112.42 (6)	B3^xxxvi^—Si1—Na1^xxii^	144.19 (12)
B2^xiii^—Mg1—Si2^xvii^	82.24 (6)	B3^i^—Si1—Na1^xxii^	94.136 (18)
B2^xiv^—Mg1—Si2^xvii^	82.24 (6)	B5^xxi^—Si1—Na1^xxii^	81.82 (5)
B2—Mg1—Si2^xvii^	112.42 (6)	Na1^xiv^—Si1—Na1^xxii^	163.65 (11)
Si3—Mg1—Si2^xvii^	52.19 (6)	B5—Si1—Na1^xxxvii^	98.18 (5)
B4^xv^—Mg1—Si2^xvii^	117.22 (8)	Si1^xxi^—Si1—Na1^xxxvii^	81.82 (5)
B4^xvi^—Mg1—Si2^xvii^	117.21 (8)	B3^xviii^—Si1—Na1^xxxvii^	144.19 (12)
B4^xvii^—Mg1—Si2^xvii^	43.85 (8)	B3^xxxvi^—Si1—Na1^xxxvii^	94.135 (18)
Si2^xv^—Mg1—Si2^xvii^	86.35 (8)	B3^i^—Si1—Na1^xxxvii^	48.18 (10)
B3^xviii^—B1—B2^iv^	108.06 (16)	B5^xxi^—Si1—Na1^xxxvii^	81.82 (5)
B3^xviii^—B1—B1^xix^	107.43 (15)	Na1^xiv^—Si1—Na1^xxxvii^	59.328 (9)
B2^iv^—B1—B1^xix^	60.40 (13)	Na1^xxii^—Si1—Na1^xxxvii^	118.01 (3)
B3^xviii^—B1—B2^xiv^	60.52 (15)	B5—Si1—Na1^xxxviii^	98.18 (5)
B2^iv^—B1—B2^xiv^	108.83 (15)	Si1^xxi^—Si1—Na1^xxxviii^	81.82 (5)
B1^xix^—B1—B2^xiv^	59.60 (13)	B3^xviii^—Si1—Na1^xxxviii^	94.137 (18)
B3^xviii^—B1—B4^ix^	59.85 (16)	B3^xxxvi^—Si1—Na1^xxxviii^	48.19 (10)
B2^iv^—B1—B4^ix^	60.63 (15)	B3^i^—Si1—Na1^xxxviii^	144.19 (12)
B1^xix^—B1—B4^ix^	108.74 (15)	B5^xxi^—Si1—Na1^xxxviii^	81.82 (5)
B2^xiv^—B1—B4^ix^	109.26 (16)	Na1^xiv^—Si1—Na1^xxxviii^	59.328 (9)
B3^xviii^—B1—Si2^i^	110.42 (15)	Na1^xxii^—Si1—Na1^xxxviii^	118.01 (3)
B2^iv^—B1—Si2^i^	136.22 (14)	Na1^xxxvii^—Si1—Na1^xxxviii^	118.01 (3)
B1^xix^—B1—Si2^i^	123.70 (8)	B5—Si1—Na1	98.18 (5)
B2^xiv^—B1—Si2^i^	107.77 (13)	Si1^xxi^—Si1—Na1	81.82 (5)
B4^ix^—B1—Si2^i^	125.94 (15)	B3^xviii^—Si1—Na1	48.19 (10)
B3^xviii^—B1—Na1	125.76 (15)	B3^xxxvi^—Si1—Na1	94.137 (18)
B2^iv^—B1—Na1	70.74 (10)	B3^i^—Si1—Na1	144.19 (12)
B1^xix^—B1—Na1	116.13 (15)	B5^xxi^—Si1—Na1	81.82 (5)
B2^xiv^—B1—Na1	173.69 (13)	Na1^xiv^—Si1—Na1	118.01 (3)
B4^ix^—B1—Na1	76.20 (11)	Na1^xxii^—Si1—Na1	59.328 (9)
Si2^i^—B1—Na1	70.23 (6)	Na1^xxxvii^—Si1—Na1	163.65 (11)
B3^xviii^—B1—B5	26.44 (12)	Na1^xxxviii^—Si1—Na1	59.329 (9)
B2^iv^—B1—B5	134.42 (13)	B5—Si1—Na1^x^	98.18 (5)
B1^xix^—B1—B5	118.8 (4)	Si1^xxi^—Si1—Na1^x^	81.82 (5)
B2^xiv^—B1—B5	60.4 (4)	B3^xviii^—Si1—Na1^x^	94.135 (18)
B4^ix^—B1—B5	80.5 (2)	B3^xxxvi^—Si1—Na1^x^	144.19 (12)
Si2^i^—B1—B5	85.06 (18)	B3^i^—Si1—Na1^x^	48.18 (10)
Na1—B1—B5	124.6 (4)	B5^xxi^—Si1—Na1^x^	81.82 (5)
B3^xviii^—B1—Na1^xx^	99.62 (14)	Na1^xiv^—Si1—Na1^x^	118.01 (3)
B2^iv^—B1—Na1^xx^	99.93 (10)	Na1^xxii^—Si1—Na1^x^	59.328 (9)
B1^xix^—B1—Na1^xx^	39.67 (11)	Na1^xxxvii^—Si1—Na1^x^	59.328 (9)
B2^xiv^—B1—Na1^xx^	39.15 (8)	Na1^xxxviii^—Si1—Na1^x^	163.65 (11)
B4^ix^—B1—Na1^xx^	139.11 (13)	Na1—Si1—Na1^x^	118.01 (3)
Si2^i^—B1—Na1^xx^	93.52 (7)	B1^i^—Si2—B1^xxix^	99.44 (13)
Na1—B1—Na1^xx^	134.55 (7)	B1^i^—Si2—B4	112.65 (8)
B5—B1—Na1^xx^	94.3 (3)	B1^xxix^—Si2—B4	112.65 (8)
B3^xviii^—B1—B5^i^	102.63 (12)	B1^i^—Si2—Si3^xv^	110.23 (7)
B2^iv^—B1—B5^i^	23.0 (2)	B1^xxix^—Si2—Si3^xv^	110.23 (7)
B1^xix^—B1—B5^i^	39.4 (2)	B4—Si2—Si3^xv^	111.12 (10)
B2^xiv^—B1—B5^i^	86.6 (2)	B1^i^—Si2—Na1^xxxiii^	166.61 (7)
B4^ix^—B1—B5^i^	72.9 (2)	B1^xxix^—Si2—Na1^xxxiii^	67.56 (6)
Si2^i^—B1—B5^i^	146.89 (9)	B4—Si2—Na1^xxxiii^	71.60 (4)
Na1—B1—B5^i^	92.17 (19)	Si3^xv^—Si2—Na1^xxxiii^	78.52 (2)
B5—B1—B5^i^	127.32 (17)	B1^i^—Si2—Na1^xxiv^	67.56 (6)
Na1^xx^—B1—B5^i^	78.9 (2)	B1^xxix^—Si2—Na1^xxiv^	166.61 (7)
B3^xviii^—B1—B5^xxi^	45.13 (19)	B4—Si2—Na1^xxiv^	71.60 (4)
B2^iv^—B1—B5^xxi^	127.76 (14)	Si3^xv^—Si2—Na1^xxiv^	78.52 (2)
B1^xix^—B1—B5^xxi^	151.4 (2)	Na1^xxxiii^—Si2—Na1^xxiv^	125.18 (3)
B2^xiv^—B1—B5^xxi^	93.9 (2)	B1^i^—Si2—Mg1^xv^	130.08 (7)
B4^ix^—B1—B5^xxi^	67.63 (14)	B1^xxix^—Si2—Mg1^xv^	130.08 (6)
Si2^i^—B1—B5^xxi^	71.88 (9)	B4—Si2—Mg1^xv^	58.69 (11)
Na1—B1—B5^xxi^	91.1 (2)	Si3^xv^—Si2—Mg1^xv^	52.42 (7)
B5—B1—B5^xxi^	33.5 (6)	Na1^xxxiii^—Si2—Mg1^xv^	63.235 (15)
Na1^xx^—B1—B5^xxi^	124.81 (18)	Na1^xxiv^—Si2—Mg1^xv^	63.235 (15)
B5^i^—B1—B5^xxi^	138.30 (13)	B1^i^—Si2—B5^i^	60.03 (17)
B3^xviii^—B1—Na1^xxii^	58.43 (12)	B1^xxix^—Si2—B5^i^	60.03 (17)
B2^iv^—B1—Na1^xxii^	59.13 (9)	B4—Si2—B5^i^	87.2 (3)
B1^xix^—B1—Na1^xxii^	105.05 (8)	Si3^xv^—Si2—B5^i^	161.7 (3)
B2^xiv^—B1—Na1^xxii^	105.67 (11)	Na1^xxxiii^—Si2—B5^i^	108.61 (12)
B4^ix^—B1—Na1^xxii^	4.27 (10)	Na1^xxiv^—Si2—B5^i^	108.61 (12)
Si2^i^—B1—Na1^xxii^	130.03 (8)	Mg1^xv^—Si2—B5^i^	145.8 (3)
Na1—B1—Na1^xxii^	79.66 (5)	B1^i^—Si2—B5^xxx^	80.55 (17)
B5—B1—Na1^xxii^	80.33 (15)	B1^xxix^—Si2—B5^xxx^	80.55 (17)
Na1^xx^—B1—Na1^xxii^	134.85 (5)	B4—Si2—B5^xxx^	51.3 (3)
B5^i^—B1—Na1^xxii^	70.00 (18)	Si3^xv^—Si2—B5^xxx^	162.4 (2)
B5^xxi^—B1—Na1^xxii^	69.77 (5)	Na1^xxxiii^—Si2—B5^xxx^	93.66 (11)
B2^xiii^—B2—B1^xxiii^	131.14 (10)	Na1^xxiv^—Si2—B5^xxx^	93.66 (11)
B2^xiii^—B2—B1^x^	111.97 (10)	Mg1^xv^—Si2—B5^xxx^	110.0 (2)
B1^xxiii^—B2—B1^x^	60.00 (14)	B5^i^—Si2—B5^xxx^	35.9 (6)
B2^xiii^—B2—B3^i^	106.92 (13)	B1^i^—Si2—Na1^xxii^	59.92 (6)
B1^xxiii^—B2—B3^i^	106.68 (18)	B1^xxix^—Si2—Na1^xxii^	59.92 (6)
B1^x^—B2—B3^i^	59.15 (14)	B4—Si2—Na1^xxii^	165.73 (10)
B2^xiii^—B2—B4^xv^	136.84 (12)	Si3^xv^—Si2—Na1^xxii^	83.15 (4)
B1^xxiii^—B2—B4^xv^	60.14 (13)	Na1^xxxiii^—Si2—Na1^xxii^	112.858 (15)
B1^x^—B2—B4^xv^	108.04 (17)	Na1^xxiv^—Si2—Na1^xxii^	112.858 (15)
B3^i^—B2—B4^xv^	106.97 (16)	Mg1^xv^—Si2—Na1^xxii^	135.57 (6)
B2^xiii^—B2—B2^xi^	120.000 (1)	B5^i^—Si2—Na1^xxii^	78.6 (3)
B1^xxiii^—B2—B2^xi^	107.39 (10)	B5^xxx^—Si2—Na1^xxii^	114.5 (2)
B1^x^—B2—B2^xi^	107.25 (10)	B1^i^—Si2—Na1^xxxix^	123.16 (7)
B3^i^—B2—B2^xi^	59.51 (9)	B1^xxix^—Si2—Na1^xxxix^	123.16 (7)
B4^xv^—B2—B2^xi^	59.65 (9)	B4—Si2—Na1^xxxix^	85.66 (9)
B2^xiii^—B2—Mg1	67.83 (6)	Si3^xv^—Si2—Na1^xxxix^	25.46 (4)
B1^xxiii^—B2—Mg1	127.40 (14)	Na1^xxxiii^—Si2—Na1^xxxix^	69.017 (15)
B1^x^—B2—Mg1	171.03 (15)	Na1^xxiv^—Si2—Na1^xxxix^	69.017 (15)
B3^i^—B2—Mg1	112.01 (15)	Mg1^xv^—Si2—Na1^xxxix^	26.96 (6)
B4^xv^—B2—Mg1	75.16 (13)	B5^i^—Si2—Na1^xxxix^	172.8 (3)
B2^xi^—B2—Mg1	66.74 (6)	B5^xxx^—Si2—Na1^xxxix^	136.9 (2)
B2^xiii^—B2—B5	71.01 (12)	Na1^xxii^—Si2—Na1^xxxix^	108.610 (15)
B1^xxiii^—B2—B5	142.0 (3)	Si3^xl^—Si3—Si2^xv^	104.62 (4)
B1^x^—B2—B5	83.9 (3)	Si3^xl^—Si3—Si2^xvi^	104.61 (4)
B3^i^—B2—B5	37.8 (2)	Si2^xv^—Si3—Si2^xvi^	113.86 (3)
B4^xv^—B2—B5	129.70 (16)	Si3^xl^—Si3—Si2^xvii^	104.61 (4)
B2^xi^—B2—B5	70.08 (13)	Si2^xv^—Si3—Si2^xvii^	113.86 (3)
Mg1—B2—B5	87.7 (3)	Si2^xvi^—Si3—Si2^xvii^	113.86 (3)
B2^xiii^—B2—Na1^xxiv^	71.62 (5)	Si3^xl^—Si3—Mg1	180.0
B1^xxiii^—B2—Na1^xxiv^	71.82 (10)	Si2^xv^—Si3—Mg1	75.38 (4)
B1^x^—B2—Na1^xxiv^	116.66 (12)	Si2^xvi^—Si3—Mg1	75.39 (4)
B3^i^—B2—Na1^xxiv^	175.02 (13)	Si2^xvii^—Si3—Mg1	75.39 (4)
B4^xv^—B2—Na1^xxiv^	76.58 (10)	Si3^xl^—Si3—Na1^xx^	118.76 (3)
B2^xi^—B2—Na1^xxiv^	125.43 (5)	Si2^xv^—Si3—Na1^xx^	136.62 (7)
Mg1—B2—Na1^xxiv^	72.08 (8)	Si2^xvi^—Si3—Na1^xx^	56.938 (18)
B5—B2—Na1^xxiv^	142.00 (18)	Si2^xvii^—Si3—Na1^xx^	56.938 (18)
B2^xiii^—B2—Na1^xxv^	113.62 (3)	Mg1—Si3—Na1^xx^	61.24 (3)
B1^xxiii^—B2—Na1^xxv^	98.99 (11)	Si3^xl^—Si3—Na1^xxv^	118.76 (3)
B1^x^—B2—Na1^xxv^	131.77 (12)	Si2^xv^—Si3—Na1^xxv^	56.938 (18)
B3^i^—B2—Na1^xxv^	92.82 (10)	Si2^xvi^—Si3—Na1^xxv^	56.940 (18)
B4^xv^—B2—Na1^xxv^	39.10 (9)	Si2^xvii^—Si3—Na1^xxv^	136.62 (7)
B2^xi^—B2—Na1^xxv^	33.32 (3)	Mg1—Si3—Na1^xxv^	61.24 (3)
Mg1—B2—Na1^xxv^	46.23 (4)	Na1^xx^—Si3—Na1^xxv^	98.79 (3)
B5—B2—Na1^xxv^	96.5 (2)	Si3^xl^—Si3—Na1^xxiv^	118.76 (3)
Na1^xxiv^—B2—Na1^xxv^	92.11 (5)	Si2^xv^—Si3—Na1^xxiv^	56.938 (18)
B2^xiii^—B2—B5^xxvi^	157.80 (15)	Si2^xvi^—Si3—Na1^xxiv^	136.62 (7)
B1^xxiii^—B2—B5^xxvi^	29.13 (9)	Si2^xvii^—Si3—Na1^xxiv^	56.940 (18)
B1^x^—B2—B5^xxvi^	69.9 (2)	Mg1—Si3—Na1^xxiv^	61.24 (3)
B3^i^—B2—B5^xxvi^	93.1 (3)	Na1^xx^—Si3—Na1^xxiv^	98.79 (3)
B4^xv^—B2—B5^xxvi^	38.8 (2)	Na1^xxv^—Si3—Na1^xxiv^	98.78 (3)
B2^xi^—B2—B5^xxvi^	78.28 (3)	Si3^xl^—Si3—Na1^xli^	36.851 (12)
Mg1—B2—B5^xxvi^	113.9 (2)	Si2^xv^—Si3—Na1^xli^	67.76 (3)
B5—B2—B5^xxvi^	130.32 (9)	Si2^xvi^—Si3—Na1^xli^	119.48 (4)
Na1^xxiv^—B2—B5^xxvi^	87.6 (2)	Si2^xvii^—Si3—Na1^xli^	119.48 (4)
Na1^xxv^—B2—B5^xxvi^	73.92 (14)	Mg1—Si3—Na1^xli^	143.149 (12)
B2^xiii^—B2—Na1^x^	111.07 (3)	Na1^xx^—Si3—Na1^xli^	155.61 (4)
B1^xxiii^—B2—Na1^x^	57.51 (9)	Na1^xxv^—Si3—Na1^xli^	97.015 (14)
B1^x^—B2—Na1^x^	3.84 (8)	Na1^xxiv^—Si3—Na1^xli^	97.015 (14)
B3^i^—B2—Na1^x^	62.97 (11)	Si3^xl^—Si3—Na1^xlii^	36.852 (12)
B4^xv^—B2—Na1^x^	107.75 (13)	Si2^xv^—Si3—Na1^xlii^	119.48 (4)
B2^xi^—B2—Na1^x^	110.53 (3)	Si2^xvi^—Si3—Na1^xlii^	119.48 (4)
Mg1—B2—Na1^x^	174.62 (10)	Si2^xvii^—Si3—Na1^xlii^	67.76 (3)
B5—B2—Na1^x^	87.0 (3)	Mg1—Si3—Na1^xlii^	143.148 (12)
Na1^xxiv^—B2—Na1^x^	112.83 (6)	Na1^xx^—Si3—Na1^xlii^	97.015 (14)
Na1^xxv^—B2—Na1^x^	133.76 (6)	Na1^xxv^—Si3—Na1^xlii^	155.61 (4)
B5^xxvi^—B2—Na1^x^	69.2 (2)	Na1^xxiv^—Si3—Na1^xlii^	97.016 (14)
B5^i^—B3—B1^xxvii^	125.38 (12)	Na1^xli^—Si3—Na1^xlii^	62.58 (2)
B5^i^—B3—B1^iv^	125.38 (12)	Si3^xl^—Si3—Na1^xliii^	36.852 (12)
B1^xxvii^—B3—B1^iv^	109.1 (2)	Si2^xv^—Si3—Na1^xliii^	119.48 (4)
B5^i^—B3—B4^xxviii^	144.1 (7)	Si2^xvi^—Si3—Na1^xliii^	67.76 (3)
B1^xxvii^—B3—B4^xxviii^	60.74 (14)	Si2^xvii^—Si3—Na1^xliii^	119.48 (4)
B1^iv^—B3—B4^xxviii^	60.74 (14)	Mg1—Si3—Na1^xliii^	143.148 (12)
B5^i^—B3—B2^i^	101.0 (6)	Na1^xx^—Si3—Na1^xliii^	97.015 (14)
B1^xxvii^—B3—B2^i^	60.33 (12)	Na1^xxv^—Si3—Na1^xliii^	97.016 (14)
B1^iv^—B3—B2^i^	109.4 (2)	Na1^xxiv^—Si3—Na1^xliii^	155.61 (4)
B4^xxviii^—B3—B2^i^	109.8 (2)	Na1^xli^—Si3—Na1^xliii^	62.58 (2)
B5^i^—B3—B2^xxix^	101.0 (6)	Na1^xlii^—Si3—Na1^xliii^	62.58 (2)
B1^xxvii^—B3—B2^xxix^	109.4 (2)		

Symmetry codes: (i) −*x*+2/3, −*y*+1/3, −*z*+1/3; (ii) *x*+1/3, *y*−1/3, *z*−1/3; (iii) −*x*+*y*+1/3, *y*−1/3, *z*−1/3; (iv) *x*−*y*+2/3, −*y*+1/3, −*z*+1/3; (v) −*x*+1, −*y*, −*z*; (vi) −*x*+*y*+1, *y*, *z*; (vii) *x*−*y*, −*y*, −*z*; (viii) *x*−*y*+2/3, *x*−2/3, −*z*+1/3; (ix) −*x*+*y*+1/3, −*x*+2/3, *z*−1/3; (x) −*y*, *x*−*y*, *z*; (xi) −*y*, −*x*, *z*; (xii) *x*, *x*−*y*, *z*; (xiii) −*x*+*y*, *y*, *z*; (xiv) −*x*+*y*, −*x*, *z*; (xv) −*x*+1/3, −*y*+2/3, −*z*+2/3; (xvi) *y*−2/3, −*x*+*y*−1/3, −*z*+2/3; (xvii) *x*−*y*+1/3, *x*−1/3, −*z*+2/3; (xviii) *x*−*y*−1/3, *x*−2/3, −*z*+1/3; (xix) −*x*+2/3, −*x*+*y*+1/3, −*z*+1/3; (xx) −*x*+*y*+2/3, −*x*+1/3, *z*+1/3; (xxi) −*x*, −*y*, −*z*; (xxii) −*x*+*y*+1, −*x*+1, *z*; (xxiii) *x*−*y*−1/3, −*y*+1/3, −*z*+1/3; (xxiv) *x*−1/3, *y*+1/3, *z*+1/3; (xxv) −*y*−1/3, *x*−*y*−2/3, *z*+1/3; (xxvi) −*x*−1/3, −*y*+1/3, −*z*+1/3; (xxvii) *y*+2/3, −*x*+*y*+1/3, −*z*+1/3; (xxviii) −*x*+4/3, −*y*+2/3, −*z*+2/3; (xxix) *y*+2/3, *x*+1/3, −*z*+1/3; (xxx) *x*+2/3, *y*+1/3, *z*+1/3; (xxxi) −*y*+2/3, *x*−*y*−2/3, *z*+1/3; (xxxii) −*y*+1, *x*−*y*, *z*; (xxxiii) −*y*+2/3, *x*−*y*+1/3, *z*+1/3; (xxxiv) −*x*+*y*+2/3, *y*+1/3, *z*+1/3; (xxxv) *y*+1/3, *x*+2/3, −*z*+2/3; (xxxvi) *y*−1/3, −*x*+*y*+1/3, −*z*+1/3; (xxxvii) *x*−1, *y*, *z*; (xxxviii) −*y*, *x*−*y*−1, *z*; (xxxix) −*x*+*y*+2/3, −*x*+4/3, *z*+1/3; (xl) −*x*, −*y*, −*z*+1; (xli) −*x*+*y*+1/3, −*x*+2/3, *z*+2/3; (xlii) −*y*+1/3, *x*−*y*−1/3, *z*+2/3; (xliii) *x*−2/3, *y*−1/3, *z*+2/3.
